# *Lactobacillus rhamnosus* GG Effect on Behavior of Zebrafish During Chronic Ethanol Exposure

**DOI:** 10.1089/biores.2015.0026

**Published:** 2016-01-01

**Authors:** Ana Claudia Reis Schneider, Eduardo Pacheco Rico, Diogo Losch de Oliveira, Denis Broock Rosemberg, Ranieli Guizzo, Fábio Meurer, Themis Reverbel da Silveira

**Affiliations:** ^1^ Programa de Pós-Graduação: Ciências em Gastroenterologia e Hepatologia, Universidade Federal do Rio Grande do Sul, Porto Alegre, Brazil.; ^2^ Programa de Pós-Graduação em Ciências da Saúde, Universidade do Extremo Sul Catarinense—UNESC, Criciúma, Brazil.; ^3^ Programa de Pós-Graduação em Ciências Biológicas: Bioquímica, Departamento de Bioquímica, ICBS, UFRGS, Porto Alegre, Brazil.; ^4^ Programa de Pós Graduação em Bioquímica Toxicológica, Centro de Ciências Naturais e Exatas, Departamento de Bioquímica e Biologia Molecular, Universidade Federal de Santa Maria, Santa Maria, Brazil.; ^5^ Universidade Federal de Ciências da Saúde de Porto Alegre, Porto Alegre, Brazil.; ^6^ Engenharia de Alimentos, Universidade Federal do Paraná (UFPR), Campus Jandaia do Sul, Jandaia do Sul, Brazil.

**Keywords:** behavior, ethanol, *Lactobacillus rhamnosus* GG, novel tank test, probiotic, zebrafish

## Abstract

Ethanol is a widely consumed drug, which acts on the central nervous system to induce behavioral alterations ranging from disinhibition to sedation. Recent studies have produced accumulating evidence for the therapeutic role of probiotic bacteria in behavior. We aimed to investigate the effect of *Lactobacillus rhamnosus* GG (LGG) on the behavior of adult zebrafish chronically exposed to ethanol. Adult wild-type zebrafish were randomly divided into four groups, each containing 15 fish. The following groups were formed: Control (C), received unsupplemented feed during the trial period; Probiotic (P), fed with feed supplemented with LGG; Ethanol (E), received unsupplemented feed and 0.5% of ethanol directly added to the tank water; and Probiotic+Ethanol (P+E), group under ethanol exposure (0.5%) and fed with LGG supplemented feed. After 2 weeks of exposure, the novel tank test was used to evaluate fish behavior, which was analyzed using computer-aided video tracking. LGG alone did not alter swimming behavior of the fish. Ethanol exposure led to robust behavioral effects in the form of reduced anxiety levels, as indicated by increased vertical exploration and more time spent in the upper region of the novel tank. The group exposed to ethanol and treated with LGG behaved similarly to animals exposed to ethanol alone. Taken together, these results show that zebrafish behavior was not altered by LGG *per se*, as seen in murine models. This was the first study to investigate the effects of a probiotic diet on behavior after a chronic ethanol exposure.

## Introduction

Alcohol dependence is a chronic and progressive disorder, which affects thousands of people around the world and may cause several physical, mental, and social impairments.^1,2^ Neurochemical alterations caused by ethanol in specific brain regions may also have effects on several neurotransmitter/neuropeptide systems, leading to behavioral alterations, which range from disinhibition to sedation and hypnosis, depending on the amount of ethanol consumed.^3^

Studies reported that intestinal microorganisms may have significant effects on the central nervous system (CNS) and on behavior.^4,5^ Chronic alcohol consumption has been known to cause dysbiosis.^6^ Much of our knowledge regarding the bidirectional communication between the nervous system and intestinal microorganisms has been produced by studies of pathogenic organisms. However, a growing number of investigations have suggested a communication between the nervous system and commensal enteric microorganisms, including probiotic bacteria.^7,8^ *Lactobacillus rhamnosus* GG (LGG) is known to modulate intestinal microbiota, preventing the increase of pathogenic bacteria and protecting the intestinal barrier during chronic ethanol exposure.^9^ Moreover, LGG metabolizes acetaldehyde, a hazardous by-product of ethanol metabolism.^10^ Acetaldehyde induces deleterious effects on the CNS, and we hypothesized if LGG could prevent behavior alterations in zebrafish chronically exposed to ethanol.

## Materials and Methods

### Animals and diet

Sixty adult wild-type (short-fin) zebrafish (*Danio rerio*) of both genders were kept in a light:dark cycle of 14:10 h and at a temperature of 28°C ± 2°C. After the acclimation period, animals were randomly assigned to one of following four groups: Control (C, *n* = 15), Probiotic (P, *n* = 15), Ethanol (E, *n* = 15), and Probiotic+Ethanol (P+E, *n* = 15). Fish were fed one of the following diets: (1) Probiotic-free diet—Control (C) and Ethanol (E) groups and (2) Feed supplemented with LGG (Culturelle^®^ Amerifit)—Probiotic (P) and Probiotic+Ethanol (P+E) groups. Both diets had a similar nutritional composition.

The viability of the LGG in the supplemented diet was determined by the standard plate-counting method in MRS (Difco), a selective medium for lactic acid bacteria. The microbiological analysis of the nonsupplemented feed showed that it was free of lactobacilli. Fish were fed twice a day until satiety.

Animals in the E and P+E groups were exposed to ethanol at a concentration of 0.5% (vol/vol) (Merck^®^ P.A), added directly into the tank water. To ensure the stability of the ethanol concentration, the water in the tanks was changed every 2 days.^11^ Same procedures were performed to C and P groups, save for the addition of ethanol to the water. Each tank (C, E, P, and P+E) contained 2 L of water and five animals each, for a total of 15 animals per group. Behavioral tests were performed after the 14th day of treatment.

Microbiological tests were performed to assess colonization by LGG in the fish gut of all groups. Fish were euthanized by hypothermal shock, and the intestines of five animals per group were entirely removed and aseptically transferred to sterile microtubes with 0.5 mL of phosphate-buffered saline, where they were macerated to prevent contact with the environment. The homogenates (20 μL) were inoculated in plates with Lactobacilli MRS Agar (Difco™) and incubated at 37°C for 2 days in microaerophilic atmosphere.

All procedures were performed according to the Brazilian legislation (Law 11.794, issued on October 8, 2008) on animal experimentation and to resolution No. 04/97 of Research Ethics Committee of the Clinical Hospital of Porto Alegre, which also concerns animal research.

### Behavioral tests

After the trial period, each fish was removed from home tank and placed individually in the novel tank.^12^ The water in the latter was kept at the same temperature of the home tank (28°C ± 2°C), however, without the addition of ethanol. The behavior of each animal was assessed in a 6-min session.^12^ Behavioral apparatus consisted of a trapezoidal tank containing 1.5 L of water and divided into three horizontal portions (lower, middle, and upper) and a camera (Microsoft Life Cam 1.1, with autofocus) connected to a computer running the software used to record animal locations and movements (ANY-maze, Stoelting Company).

Experimental procedures were similar for all animals, and behavioral tests were always performed in the same room to ensure similar environment and lighting across all trials. After each test, the water in the novel tank was changed. Fish behavior was examined by measuring the following variables: total distance travelled (m), mean speed (m/s), time mobile (s), and number of transitions between areas and time spent in each location (lower, middle, and upper).

### Statistical analyses

Behavioral data are expressed as mean ± standard error of the mean. The number of transitions between areas in the tank and the total swimming time (time mobile) was analyzed using a two-way ANOVA and Tukey as *post hoc* test. Analyses were performed using the GraphPad Prism software, version 6.0 for Windows (GraphPad Software). Results were considered significant at *p* ≤ 0.05.

## Results

### Microbiological analyses

The incubated plates of the intestinal samples from the groups P and P+E showed growth of LGG colonies, but no colonies grew in plates of C and E groups. The phenotypic characterization of the colonies cultured in plates of P and P+E groups was compatible with the colonies obtained from the positive control.

### Total distance travelled, mean velocity, and total swimming time

**FIG. 1.**
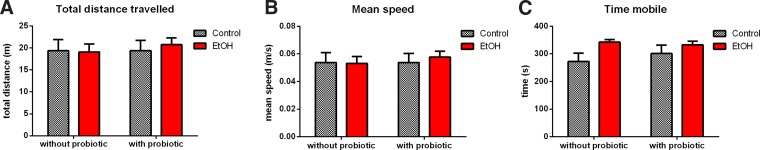
Evaluation of the locomotor activity parameters in the novel tank test: total distance travelled **(A),** mean speed **(B),** and time mobile **(C)** did not significantly differ between experimental groups. Statistical analysis was performed by two-way ANOVA and Tukey as *post hoc*; *n* = 15 animals/group. Data are expressed as mean ± SEM, and *p* ≤ 0.05 was considered statistically significant.

The absence of significant alterations in the locomotor activities shown in Figure 1—total distance travelled (A), mean speed (B), and time mobile (C)—demonstrated that neither ethanol nor probiotic exposure led to sedation or toxicity.

### Effects of ethanol exposure

Swimming patterns of fish exposed to ethanol were significantly different from those observed in the remaining groups. In most tests, the exploratory behavior observed in animals exposed to ethanol was distinct from that recorded in the control and probiotic groups. No interactions were observed between ethanol and probiotic exposure.

**FIG. 2.**
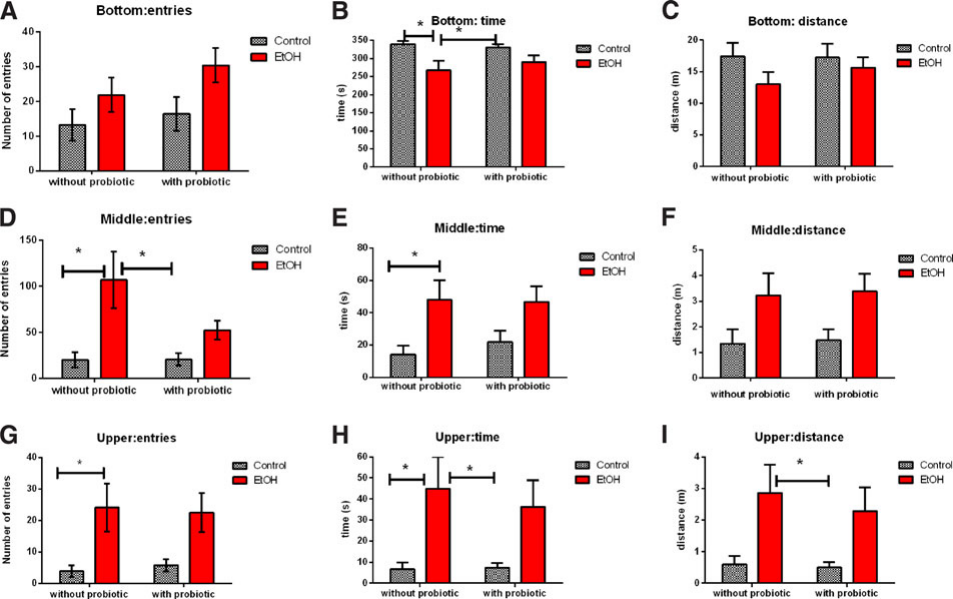
Exploratory activity of zebrafish in the novel tank test: ethanol caused the main effect in fish behavior related to the number of entries **(D, G)**; time spent in each area **(B, E, H)**; and distance travelled **(I)**. P+E group did not show any statistical difference compared to E, C, and P groups. Animals exposed to ethanol explored the upper level of the tank significantly more than the other groups and spent more time in this zone of the tank **(G, H, I)**. **(A, C, F)** There were no statistically significant differences between groups. Data are expressed as mean ± SEM, and *p* ≤ 0.05 was considered statistically significant. *Significant difference between groups. Statistical analysis was performed by two-way ANOVA followed by Tukey as post hoc; *n* = 15 animals/group.

Animals of E group spent less time in the bottom portion of the tank than control fish and probiotic-alone groups (Fig. 2B; F(1, 55) = 11.03; *p* = 0.0016). Ethanol exposure was found to have a main effect on this variable, although it had no impact on the number of entries and distance travelled in the bottom of the tank (Fig. 2B, C). The E group behavior differed from C and P groups in the middle portion of the tank (number of entries: F(1, 54) = 14.38; *p* = 0.009; time spent in area: F(1, 56) = 10.87; *p* = 0.0017). These results are presented in Figure 2D and 2E.

The analysis of swimming behaviors in the upper portion of the tank revealed that animals exposed to ethanol spent more time in this location. Ethanol exposure influenced on the number of transitions (F(1, 56) = 12.66; *p* = 0.0008), time spent (F(1, 56) = 11.23; *p* = 0.0014), and distance travelled in the upper level of the tank (F(1, 56) = 11.09; *p* = 0.0015) (Fig. 2G, H, I).

The P+E group behavior did not present any significant difference in comparison to E, C, and P groups. Animals of P and C groups behaved similarly in all tests performed. No interactions were observed between the probiotic diet and ethanol exposure group and only the latter appeared to have an impact on fish behavior.

## Discussion

Although many different experimental protocols have been used to assess anxiety in fish, the novel tank test is one of the most popular methods to evaluate this variable.^12–15^ In this procedure, anxiety is defined as reduced environmental exploration and fewer transitions between areas in the tank.^13–15^ When animals are placed in new environments, they initially swim in a vertical direction, exploring the upper areas of the tank. This behavior is associated with adaptation to the new setting. Greater vertical swimming, assessed by the time spent in the upper levels of the tank and the number of transitions to this location, is considered indicative of lower anxiety levels.^13–16^ Our results showed that ethanol exposure induces a significant change in behavioral indicators of anxiety in zebrafish. Similar results were related by Cachat, Egan, and Wong, despite the differences in experimental time and concentrations of ethanol exposure.^14,15–17^

Ethanol acted as an anxiolytic agent in group E, although no further behavioral alterations were observed in animals who received the probiotic LGG in addition to ethanol (P+E). As can be observed in Figure 2, the behavior of animals of P+E group was similar to that exposed to E alone, although it did not differ significantly from that of the control group. This finding may be explained by the presence of alcohol dehydrogenase (ADH) and aldehyde dehydrogenase (ALDH) in lactobacilli.^10^ Acetaldehyde is the first product of ethanol oxidation in the body and it affects the activity of several neurotransmitters and, consequently, can contribute to the behavioral alterations associated with ethanol intake.^18,19^ Two different studies have shown that lactobacilli upregulate the activity of ADH and ALDH, increasing ethanol oxidation by bacterial dehydrogenases.^20,21^ The resulting acetaldehyde is oxidized by ALDH into acetate, decreasing ethanol concentrations in the blood. Perhaps the absence of behavioral differences between P+E and C groups could be explained by a decrease of ethanol concentration in the blood.

## Conclusions

Zebrafish fed with LGG alone did not show any behavioral alterations. The anxiolytic effects of ethanol were not significantly altered by LGG intake.

## Abbreviations Used

ADH: alcohol dehydrogenase
ALDH: aldehyde dehydrogenase
CNS: central nervous system
LGG: *Lactobacillus rhamnosus* GG

## References

[1] Gramenzi A, Caputo F, Biselli M, et al. Review article: alcoholic liver disease—pathophysiological aspects and risk factors. Aliment Pharmacol Ther. 2006;24:1151–1161 17014574 10.1111/j.1365-2036.2006.03110.x

[2] Gunzerath L, Faden V, Zakhari S, et al. National Institute on Alcohol Abuse and Alcoholism report on moderate drinking. Alcohol Clin Exp Res. 2004;28:829–847 15201626 10.1097/01.alc.0000128382.79375.b6

[3] Spanagel R Alcoholism: a systems approach from molecular physiology to addictive behavior. Physiol Rev. 2009;89:649–705 19342616 10.1152/physrev.00013.2008

[4] Cryan JF, O'Mahony SM The microbiome-gut-brain axis: from bowel to behavior. Neurogastroenterol Motil. 2011;23:187–192 21303428 10.1111/j.1365-2982.2010.01664.x

[5] Cryan JF, Dinan TG Mind-altering microorganisms: the impact of the gut microbiota on brain and behaviour. Nat Rev Neurosci. 2012;13:701–712 22968153 10.1038/nrn3346

[6] Keshavarzian A, Fahradi A, Forsyth CB, et al. Evidence that chronic alcohol exposure promotes intestinal oxidative stress, intestinal hyperpermeability and endotoxemia prior to development of alcoholic steatohepatitis in rats. J Hepatol. 2009;50:538–547 19155080 10.1016/j.jhep.2008.10.028 PMC2680133

[7] Messaoudi M, Lalonde R, Violle N, et al. Assessment of psychotropic-like properties of a probiotic formulation (*Lactobacillus helveticus R 0052 and Bifidobacterium longum R 0175*) in rats and human subjects. Br J Nutri. 2011;105:755–764 10.1017/S0007114510004319 20974015

[8] Rao AV, Bested AC, Beaulne TM, et al. A randomized, double-blind, placebo-controlled pilot study of a probiotic in emotional symptoms of chronic fatigue syndrome. Gut Pathog. 2009;1:6 19338686 10.1186/1757-4749-1-6 PMC2664325

[9] Bull-Otterson L, Feng W, Kirpich I, et al. Metagenomic analyses of alcohol induced pathogenic alterations in the intestinal microbiome and the effect of *Lactobacillus rhamnosus* GG treatment. PLoS One. 2013;8:e53028 23326376 10.1371/journal.pone.0053028 PMC3541399

[10] Nosova T, Jousimies-Somer H, Jokelainen K, et al. Acetaldehyde production and metabolism by indigenous and probiotic *Lactobacillus* and *Bifidobacterium* strains. Alcohol Alcohol. 2000;35:561–568 11093962 10.1093/alcalc/35.6.561

[11] Schneider ACR, Machado ABMP, de Assis AM, et al. Effects of *Lactobacillus rhamnosus* GG on hepatic and serum lipid profiles in zebrafish exposed to ethanol. Zebrafish. 2014;11:371–378 24987799 10.1089/zeb.2013.0968

[12] Rosemberg DB, Braga MM, Rico EP, et al. Behavioral effects of taurine pretreatment in zebrafish acutely exposed to ethanol. Neuropharmacology. 2012;63:613–623 22634362 10.1016/j.neuropharm.2012.05.009

[13] Stewart A, Gaikwad S, Kyzar E, et al. Modeling anxiety using adult zebrafish: a conceptual review. Neuropharmacology. 2012;62:135–143 21843537 10.1016/j.neuropharm.2011.07.037 PMC3195883

[14] Cachat J, Stewart A, Grossman L, et al. Measuring behavioral and endocrine responses to novelty stress in adult zebrafish. Nat Protoc. 2010;5:1786–1799 21030954 10.1038/nprot.2010.140

[15] Wong K, Elegante M, Bartels B, et al. Analyzing habituation responses to novelty in zebrafish (*Danio rerio*). Behav Brain Res. 2010;208:450–457 20035794 10.1016/j.bbr.2009.12.023

[16] Mathur P, Guo S Differences of acute versus chronic ethanol exposure on anxiety-like behavioral responses in zebrafish. Behav Brain Res. 2011;219:234–239 21255611 10.1016/j.bbr.2011.01.019 PMC3062742

[17] Egan RJ, Bergner CL, Hart PC, et al. Understanding behavioral and physiological phenotypes of stress and anxiety in zebrafish. Behav Brain Res. 2009;205:38–44 19540270 10.1016/j.bbr.2009.06.022 PMC2922906

[18] Zenki KC, Mussulini BH, Rico EP, et al. Effects of ethanol and acetaldehyde in zebrafish brain structures: an in vitro approach on glutamate uptake and on toxicity-related parameters. Toxicol In Vitro. 2014;28:822–828 24681127 10.1016/j.tiv.2014.03.008

[19] Rico EP, Rosemberg DB, Senger MR, et al. Ethanol and acetaldehyde alter NTPDase and 5′-nucleotidase from zebrafish brain membranes. Neurochem Int. 2008;52:290–296 17698255 10.1016/j.neuint.2007.06.034

[20] Park JH, Kim Y, Kim SH Green tea extract (*Camellia sinensis*) fermented by *Lactobacillus fermentum* attenuates alcohol-induced liver damage. Biosci Biotechnol Biochem. 2012;76:2294–2300 23221715 10.1271/bbb.120598

[21] Qing L, Wang T Lactic acid bacteria prevent alcohol-induced steatohepatitis in rats by acting on the pathways of alcohol metabolism. Clin Exp Med. 2008;8:187–191 18813871 10.1007/s10238-008-0002-4

